# Heat‐Induced Toxicants: Sensory Appeal Distracts Consumers' Attention From Potential Toxicological Risks of Thermally Processed Foods

**DOI:** 10.1002/fsn3.71766

**Published:** 2026-04-13

**Authors:** Joachim Dotto Matondo, Abdulsudi Issa‐Zacharia

**Affiliations:** ^1^ Department of Food Science and Agro‐Processing Sokoine University of Agriculture Morogoro Tanzania

**Keywords:** carcinogenicity, heat‐induced contaminants, Millard reaction, neurotoxicity, risk mitigation, thermal processing

## Abstract

Thermal processing of foods creates an appealing sensory experience of golden‐brown hues, flavorful aromas, and crispy textures; yet the very Maillard reactions, caramelization, and lipid degradation that produce these desirable attributes simultaneously generate hazardous heat‐induced toxicants. This review synthesizes current evidence on acrylamide, heterocyclic aromatic amines, polycyclic aromatic hydrocarbons, and advanced glycation end‐products, demonstrating how these contaminants are associated with carcinogenicity, neurotoxicity, and metabolic dysfunction through oxidative stress, inflammation, and genotoxicity. We evaluate advanced chromatographic and spectroscopic detection methods alongside strategic mitigation approaches spanning raw material selection, enzymatic pre‐treatment, and processing optimization. This review uniquely contributes a systematic analysis of the intrinsic biochemical entanglement between desirable sensory properties and toxicant generation, demonstrating through integrated evidence that the Maillard reaction constitutes a critical branching point where strategic intervention can simultaneously suppress multiple contaminants. Ultimately, reconciling this fundamental conflict between palatability and long‐term health safety remains a formidable challenge, necessitating future research focused on producing safer thermally processed foods without compromising consumer acceptance.

## Graphical abstract

The central paradox of thermal processing of foods. (A) The application of heat (e.g., frying, baking, grilling) to food initiates complex chemical reactions, most notably the Maillard reaction and thermal degradation of protein, lipids, and carbohydrate. (B) These reactions create a desirable sensory experience characterized by enticing aromas, golden‐brown colors, and palatable textures that drives consumer preference. (C) However, the very same biochemical pathways simultaneously generate a suite of hazardous HITs, including acrylamide, heterocyclic aromatic amines (HAAs), polycyclic aromatic hydrocarbons (PAHs), and advanced glycation end‐products (AGEs). D: This duality creates a significant conflict between short‐term sensory appeal and long‐term health safety.

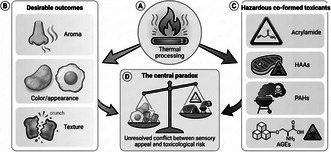

## 1 Background

The aroma of freshly baked bread, the golden crust of a roasted potato, and the rich flavor of grilled meat are universally cherished sensory experiences, deeply embedded in culinary traditions and consumer expectations. These desirable attributes are the symbol of thermal processing, a cornerstone of modern food manufacturing that ensures safety, extends shelf life, and creates palatable textures (Obando and Figueroa 2024; Shi et al. 2025). However, a growing body of evidence reveals a troubling paradox: the very chemical reactions that generate these appealing qualities simultaneously produce a spectrum of hazardous compounds known as heat‐induced toxicants (HITs) (Hu et al. 2025; Liu et al. 2022; Rasool et al. 2025). This inherent duality of thermal processing, where sensory perfection is chemically inseparable from toxicological risk, forms the central conflict examined in this review.

The formation of HITs is an inevitable consequence of high‐temperature cooking. Processes such as the Maillard reaction, sugar caramelization, and lipid degradation, fundamental to developing flavor and color, also generate potent contaminants including acrylamide, heterocyclic aromatic amines (HAAs), polycyclic aromatic hydrocarbons (PAHs), and advanced glycation end‐products (AGEs) (Bolchini et al. 2025; Wang, Wang, et al. 2023). Acrylamide, classified by the International Agency for Research on Cancer (IARC) as a probable human carcinogen (Group 2A), forms predominantly in starchy foods cooked above 120°C via the reaction of asparagine with reducing sugars (IARC 2023; Liu et al. 2022). The scale of this issue is illustrated by occurrence data; French fries can contain over 1000 μg/kg of acrylamide, while potato chips can range from 200 to 3500 μg/kg, leading to disproportionately high exposure levels in children (Başaran et al. 2023; Perera et al. 2021; Vignesh et al. 2024).

Simultaneously, the high‐temperature processing like frying or grilling of meats generates HAAs, such as 2‐amino‐1‐methyl‐6‐phenylimidazo[4,5‐b]pyridine (PhIP, a carcinogen linked to colon and breast cancer) and 2‐amino‐3,8‐dimethylimidazo[4,5‐f]quinoxaline (MeIQx, a known mutagen and carcinogen), at temperatures between 150°C and 250°C (Aoudeh et al. 2024; Wang, Chu, et al. 2023). The toxicological potency of these compounds is alarming; specific HAAs exhibit over 1000‐fold greater mutagenic activity than aflatoxin B1 in certain bacterial assays (Aoudeh et al. 2024). Furthermore, grilling, barbecuing, and smoking fats generate PAHs, including the known human carcinogen benzo[a]pyrene (BaP, Group 1), alongside benz[a]anthracene (BaA), benzo[b]fluoranthene (BbF), and benzo[k]fluoranthene (BkF) (Chiang et al. 2022; da Silva et al. 2024; IARC 2023). The regulatory framework struggles to contain this risk; while the European Union sets a strict limit of 2.0 μg/kg for BaP in smoked meats and fish, analyses of smoked sausages report total PAH levels reaching 108.24 μg/kg, a concentration that far exceeds the established thresholds (da Silva et al. 2024; EC 2011). Even seemingly benign staples contribute to the burden: coffee preparation increases hydroxymethylfurfural (HMF), a genotoxic compound, by 224.75%, reaching 362.05 mg/kg (Farag et al. 2020; Tang et al. 2024). Similarly, heating polyunsaturated fatty acids (PUFAs) in cooking oils can trigger a 10‐fold increase in the formation of toxic aldehydes like acrolein and 4‐hydroxy‐2‐nonenal (4‐HNE), both linked to respiratory damage and atherosclerosis (Xu et al. 2025; Zhuang et al. 2022).

The critical challenge, and the focus of this review, is that the very sensory cues consumers actively seek and trust are direct indicators of this chemical hazard. The development of roasted, nutty flavors from alkylpyrazines in coffee, meat, and chocolate relies on pathways that inevitably co‐produce HAAs and acrylamide (Cascos et al. 2024; Park and Choi 2025; Shi et al. 2025). Kinetic studies confirm a power law correlation between browning intensity and acrylamide generation, meaning the visually appealing golden‐brown hue that consumers use to judge perfect doneness simultaneously signals the point of maximal toxicant formation (Chen, Jiao, et al. 2022; El Hosry et al. 2025).

Given the ubiquitous presence of HITs in common foods, from crisps and toast to coffee and barbecued meats, the cumulative effect of chronic, low‐dose dietary exposure constitutes a significant public health concern. This review therefore synthesizes current knowledge on the formation pathways and occurrence of major HITs. It examines the epidemiological and toxicological evidence linking chronic dietary exposure to cancer, neurotoxicity, and metabolic disorders. Finally, it evaluates the efficacy of advanced detection methods and strategic mitigation approaches, from raw material selection to process optimization, providing a comprehensive overview of the conflict between sensory appeal and long‐term health safety in our modern food supply.

## 2 Methods and Materials

A comprehensive literature search was conducted using Web of Science, Scopus, Google Scholar, and PubMed to identify peer‐reviewed studies on heat‐induced toxicants in thermally processed foods. The search strategy employed Boolean operators and a range of terms, including: (“heat‐induced toxicant” OR “processing contaminant” OR “process‐induced toxicant”) AND (“Maillard reaction” OR “acrylamide” OR “heterocyclic aromatic amine” OR “HAA” OR “polycyclic aromatic hydrocarbon” OR “PAH” OR “advanced glycation end‐product*” OR “AGE” OR “furan” OR “hydroxymethylfurfural” OR “acrolein”) AND (“sensory propert” OR “flavor*” OR “aroma” OR “color” OR “texture” OR “browning”) AND (“formation pathway” OR “occurrence” OR “health” OR “carcinogenicity” OR “neurotoxicity” OR “mitigation” OR “detection”). We further refined results using database‐specific filters, citation chaining (forward and backward), and manual screening of key journals in food science and toxicology to capture seminal and recent articles (2018–2025). This process ensured comprehensive coverage of formation pathways, health implications, and mitigation strategies for major toxicants. For data synthesis, qualitative thematic analysis was performed using *ATLAS.ti* software (v7.5.16 for Windows) to systematically identify and collate recurring themes, such as the co‐formation of sensory compounds and toxicants, underlying biochemical mechanisms, and intervention strategies. All figures were created using *EdrawMax* software (v12.0.6 for Windows), with graphical elements sourced exclusively from the default image library of the software to ensure consistency and visual clarity.

## 3 Occurrences and Types of Heat‐Induced Toxicants

### 3.1 Maillard Reactions

The Maillard reaction, a complex network of non‐enzymatic reactions between amino acids and reducing sugars, functions as a dual‐purpose pathway in thermally processed foods, simultaneously generating desirable sensory properties and an array of undesirable toxicants through shared chemical routes (Bolchini et al. 2025; Liu et al. 2022). This duality originates from common reactive intermediates; the initial condensation forms a Schiff base, which rearranges into Amadori products before decomposing into highly reactive α‐dicarbonyl compounds (El Hosry et al. 2025). Subsequently, these α‐dicarbonyls act as critical branching points, undergoing further reactions that yield both flavor‐rich heterocycles and hazardous substances (El Hosry et al. 2025). Notably, acrylamide, a probable human carcinogen and neurotoxicant, is predominantly generated in starchy foods like potatoes and cereals cooked above 120°C via the reaction of asparagine with carbonyl sources (Augustine and Bent 2022; Mirza Alizadeh et al. 2025). Its formation pathway involves Schiff base decarboxylation to form azomethine ylide, the direct precursor (Augustine and Bent 2022). Moreover, the potential for formation is significantly influenced by food composition; for instance, precursor concentration varies, with raw rye containing 829 mg/kg of asparagine and wheat containing 292 mg/kg, directly impacting the toxicological profile of baked goods (Qi et al. 2025). Concurrently, free amino acids and creatine react at temperatures between 150°C and 250°C to form HAAs, a class of potent mutagens and carcinogens including PhIP, MeIQx, and 2‐Amino‐9H‐pyrido[2,3‐b]indole (AαC), frequently detected in grilled and fried meats (Kwon et al. 2025; Tamanna and Mahmood 2015). Furthermore, the reaction propagates advanced AGEs, such as Nε‐carboxymethyllysine (CML) and Nε‐carboxyethyllysine (CEL), which form from lysine residues and α‐dicarbonyls like glyoxal and are associated with chronic diseases including diabetes and cardiovascular disorders (Chen, Jiao, et al. 2022; El Hosry et al. 2025). Critically, the co‐formation of these toxicants is highly interdependent, as kinetic studies reveal that amino acids compete for limited reactive intermediates; for example, lysine can compete with asparagine for α‐dicarbonyl compounds, thereby simultaneously suppressing AGEs and acrylamide formation while potentially enhancing the generation of other heterocyclic amines through alternative pathways like the Pictet–Spengler condensation (Chen, Jiao, et al. 2022; El Hosry et al. 2025). This intricate competition emphasizes that the Maillard reaction constitutes a complex chemical system wherein the formation of one toxicant directly influences the yield of another, creating a significant challenge where the very pathways producing appealing nutty, caramel‐like, and roasted aromas also establish a concerning toxicological signature (Bolchini et al. 2025; Liu et al. 2022). The sources, example foods, and associated health risks for these major toxicants are summarized in Table 1.

**TABLE 1:** Common heat‐induced toxicants: Sources, precursors, example foods, and health risks.

Toxicant	Formation pathway	Key precursor	Example food	Key health risk	IARC^a^	References
Acrylamide	Maillard reaction	Asparagine; reducing sugars	French fries, potato crisps, coffee, toasted bread	Neurotoxicity, carcinogenicity	2A	Qi et al. (2025)
AGEs	Maillard reaction	Lysine; α‐dicarbonyl	Roasted meats/sausages, hard cheese, butter; bakery products	Oxidative stress; inflammation	Not classified	Chen, Jiao, et al. (2022)
Acrolein	Lipid degradation	Glycerol; PUFAs	Heated oils, deep fried fat foods; some alcoholic drinks; distilled spirits	Irritation; atherosclerosis	3	Lim and Shin (2025)
HAAs (IQ, MeIQ, MeIQx, PhIP)	Maillard reaction; pyrolysis	Creatine; amino acids	Pan‐fried meats, grilled fish/meat, barbecued meat	Multisite carcinogenicity	2A for IQ; 2B for MeIQ, MeIQx, PhIP	Kwon et al. (2025)
PAHs (BaP, BaA, BbF, BkF, other PAHs)	Incomplete combustion; pyrolysis	Fats; organic matter	Smoked foods, grilled meats, charcoal‐roasted foods	Carcinogenicity; mutagenicity	1 for BaP; 2B for BaA, BbF, BkF; 2A or 2B for other PAHs	Rigi et al. (2025)
Furan	Sugar caramelization; Maillard reaction	Sugars, amino acids, ascorbic acid	Canned/jarred meats, coffee, heated foods	Liver toxicity; carcinogenicity	2B	Goswami et al. (2025)
HMF	Sugar dehydration; Maillard reaction	Hexose sugars	Coffee, caramels, dried fruits, bread crusts, concentrates	Cytotoxicity; genotoxicity (in vitro)	Not classified	Farag et al. (2020)

Abbreviations: AGEs, advanced glycation end‐products; BaA, benz[a]anthracene; BaP, benzo[a]pyrene; BbF, benzo[b]fluoranthene; BkF, benzo[k]fluoranthene; HAA, heterocyclic aromatic amines; HMF, hydroxymethylfurfural; IQ, 2‐amino‐3‐methyl‐3H‐imidazo[4,5‐f]quinoline; MeIQ, 4‐methyl‐2‐amino‐3‐methylimidazo‐[4,5‐f]quinoline; MeIQx, 2‐samino‐3,8‐dimethylimidazo[4,5‐f]quinoxaline; PAHs, polycyclic aromatic hydrocarbons; PhIP, 2‐amino‐1‐methyl‐6‐phenylimidazo[4,5‐b]pyridine; PUFAs, polyunsaturated fatty acids.

^a^ *IARC classification*: Group 1 (carcinogenic to humans), Group 2A (probably carcinogenic to humans), Group 2B (possibly carcinogenic to humans), Group 3 (not classifiable as to its carcinogenicity to humans), Group 4 (probably not carcinogenic to humans) (IARC 2023).

### 3.2 Sugar Dehydration and Caramelization

Distinct from the Maillard pathway, the thermal processing of carbohydrate‐rich foods initiates sugar dehydration and caramelization, a dualistic process that concurrently develops appealing sensory compounds and generates a range of hazardous compounds, thereby creating a complex risk–benefit landscape. These non‐enzymatic browning reactions predominantly yield furanic compounds, with furan and hydroxymethylfurfural (HMF) identified as primary toxicological concerns (Chan et al. 2025; Goswami et al. 2025). Furan, a volatile heterocyclic compound originates from multiple parallel pathways involving the thermal decomposition of ascorbic acid, the dehydration of reducing sugars, and the oxidation of polyunsaturated fatty acids, a complexity highlighted in diverse food matrices (Batool et al. 2023; Seok et al. 2015). Its formation escalates with thermal intensity, as evidenced in vegetable oils where soybean oil, due to its high linolenic acid content, generated increasing furan concentrations when heated from 170°C to 190°C (Emektar et al. 2022). Concurrently, HMF emerges as a ubiquitous intermediate and reliable chemical marker for heat intensity, formed via the acid‐catalyzed dehydration of hexose sugars or through Maillard reaction pathways (Chan et al. 2025; Farag et al. 2020). Quantitative levels of these toxicants are governed by processing parameters; for instance, deep‐frying breaded fish products in olive oil yielded 30.36 μg/g of total furanic compounds, a level threefold higher than the 9.99 μg/g detected in oven‐baked batches (Pérez‐Palacios et al. 2013). Consequently, commonplace items like baked goods, canned products, and roasted coffee become major dietary vectors. Specific occurrences include wheat bread with cranberries (210 mg/kg HMF), breakfast cereals like honey wheat loops (85.09 mg/kg HMF), and coffee, where HMF levels can surge by 224.75% post‐preparation, reaching 362.05 mg/kg (Farag et al. 2020). Beyond furan and HMF, the caramelization process yields other toxic sugar degradation products such as furfural, 2‐pentylfuran, and the polymeric constituents of caramel colors—namely caramelan, caramelen, and caramelin (EFSA Panel on Additives and Products or Substances used in Animal Feed (FEEDAP) et al. 2023; Sengar and Sharma 2014). The primary toxicological concern for HMF revolves around its metabolic conversion to 5‐sulfoxymethylfurfural (SMF), a genotoxic intermediate capable of binding to DNA, which underpins regulatory scrutiny leading to suggested intake limits of 1.6 mg HMF per day and specific limits of 40 mg/kg for honey (Chan et al. 2025). Therefore, mitigating these toxic compounds without compromising the sensory qualities that define product acceptability remains a formidable challenge for food scientists and technologists.

### 3.3 Lipid Thermal Degradation

**FIGURE 1:**
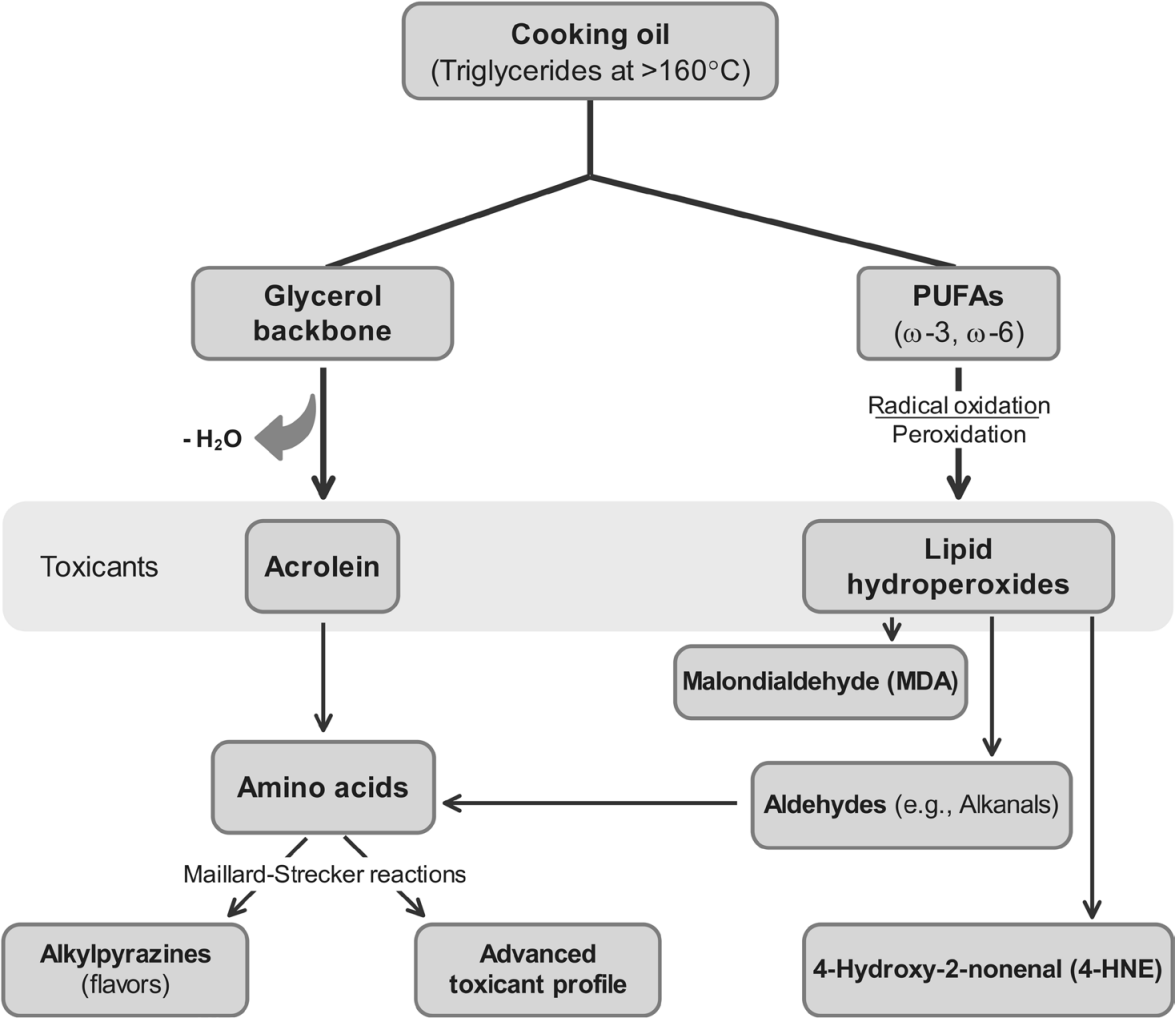
Pathways of lipid thermal degradation generating toxic carbonyl compounds. The schematic illustrates the formation of primary toxicants, particularly acrolein, from the glycerol backbone and secondary lipid oxidation products (LOPs) such as malondialdehyde (MDA) and 4‐hydroxy‐2‐nonenal (4‐HNE) from the peroxidation of polyunsaturated fatty acids (PUFAs). The intersection of these lipid‐derived carbonyls with the Maillard reaction and Strecker degradation contributes to both flavor (alkylpyrazines) and additional toxicant burden.

In parallel, thermal processing of lipids initiates complex oxidative degradation, breaking down triglycerides generating a spectrum of hazardous compounds that subvert both nutritional integrity and consumer safety. As temperatures increase through the critical 100°C to 200°C frying window, lipid oxidation products (LOPs) surge significantly, with PUFA‐rich oils such as soybean and sunflower displaying marked vulnerability; they generate substantially higher toxic aldehyde loads than their saturated counterparts like palm or olive oil (Hu et al. 2024; Zhuang et al. 2022). This degradation yields oxidized triglycerides (oxTGs), diglycerides, and free fatty acids, basically undermining nutritional integrity (Hu et al. 2024). A primary toxicant, acrolein, forms from glycerol backbone dehydration; this highly irritating α,β‐unsaturated aldehyde is exacerbated during frying and is implicated in respiratory and ocular damage (Koszucka and Nowak 2019; Mirza Alizadeh et al. 2025). Acrolein can further manifest through alternative routes, including pyrolysis of carbohydrates; thermal degradation of amino acids such as methionine and cystathionine; and oxidation of PUFAs, emphasizing its prevalent formation across diverse food matrices (Mirza Alizadeh et al. 2025; Zhuang et al. 2022). Concurrently, radical‐mediated peroxidation attacks PUFAs, generating a milieu of secondary LOPs (Zhang et al. 2021). These LOPs include saturated and unsaturated aldehydes, ketones, carboxylic acids, and higher polymers, as well as a cytotoxic repertoire of secondary carbonyls (Grootveld 2022). Among these secondary carbonyls, malondialdehyde (MDA) and 4‐HNE emerge prominently, with 4‐HNE recognized as a potent genotoxin and biomarker of oxidative stress that adducts DNA and proteins (Hu et al. 2024; Zhang et al. 2021). The schematic in Figure 1 illustrates these interconnected pathways of lipid thermal degradation, depicting the formation of primary toxicants such as acrolein from the glycerol backbone alongside secondary LOPs including MDA and 4‐HNE from the peroxidation of PUFAs.

Furthermore, in the presence of chloride ions, lipid degradation pathways intersect to form chlorinated progenitors such as 3‐monochloropropane‐1,2‐diol (3‐MCPD), a processing contaminant with recognized nephrotoxic and carcinogenic potential (Koszucka and Nowak 2019; Mirza Alizadeh et al. 2025). The chemical landscape is further complicated by synergistic interactions between lipid peroxidation and the Maillard reaction. Lipid‐derived carbonyls, including alkanals and alkenals, can react with amino acids to form alkylpyrazines; these heterocyclic compounds contribute desirable aromas and flavors in related foods but effectively override the underlying toxicological risk (Delgado et al. 2016; Koszucka and Nowak 2019). This co‐oxidation significantly expands the profile of thermal toxicants to include HAAs and PAHs (Grootveld 2022; Koszucka and Nowak 2019). The physiological implications are severe, as these chemically reactive α,β‐unsaturated aldehydes are absorbed systemically and are associated with mutagenicity, genotoxicity, and pro‐inflammatory responses, posing a critical safety concern where desirable sensory compounds co‐form with health‐compromising toxicants (Grootveld 2022; Zhang et al. 2021).

### 3.4 Protein Pyrolysis and Incomplete Combustion of Fats Produce PAHs

Polycyclic aromatic hydrocarbons (PAHs) represent a major class of HITs, forming primarily through protein pyrolysis and the incomplete combustion of organic matter during thermal food processing such as grilling, barbecuing, and smoking. These hazardous organic compounds, characterized by two or more fused benzene rings, derive their chemical stability from a dense cloud of π electrons within their aromatic structures (Patel et al. 2020). Their formation initiates with the thermal fragmentation of fats, proteins, and carbohydrates into smaller, highly reactive free radicals and molecules like ethylene and acetylene at temperatures typically exceeding 200°C (Das et al. 2023; Lin et al. 2024). Subsequently, these radical fragments undergo complex recombination and cyclization through elusive mechanisms including propargyl recombination, hydrogen abstraction and acetylene addition (HACA), and phenyl addition cyclization (PAC), which collectively facilitate benzene ring formation and eventual condensation into high molecular weight polycyclic structures (Das et al. 2023). A critical amplification pathway occurs when fat drips onto open flames or hot heating elements during grilling; this accelerates pyrolysis, generating volatile PAHs that are carried by smoke and deposited onto food surfaces (Duedahl‐Olesen and Ionas 2022). Concurrently, recent studies confirm that the Maillard reaction between amino acids like proline and reducing sugars under high temperature conditions (125°C–160°C for 1.5–2 h) generates melanoidins (Wang et al. 2025). Pyrolysis of these proline‐specific melanoidins, particularly at temperatures exceeding 200°C, promotes PAH formation via radical‐mediated pathways like hydrogen‐abstraction acetylene‐addition (HACA), with pyrroles and other heterocycles acting as crucial intermediates that serve as potent PAH precursors during pyrolysis (Liu et al. 2025; Wang et al. 2025). Consequently, the resulting PAH contamination level is highly variable, influenced by cooking temperature, duration, distance from the heat source, and food composition, with charcoal grilling generating significantly higher concentrations than gas grilling or electric oven roasting (Rigi et al. 2025). The key toxicants from this and other major formation pathways are consolidated in Table 1. Among the numerous PAHs identified, benzo[a]pyrene (BaP) stands out as a key representative and probable human carcinogen, although regulatory focus has shifted toward monitoring a panel of four (PAH4: benz[a]anthracene, chrysene, benzo[b]fluoranthene, and BaP) or eight (PAH8) compounds for a more comprehensive risk assessment (Sahin et al. 2020; Zelinkova and Wenzl 2015). Quantitative data illustrate this contamination, with studies reporting total PAH (Σ16PAH) levels ranging from 4.42 μg/kg in grilled chicken to 7.26 μg/kg in grilled fish (Sahin et al. 2020). Alarmingly, a study of Brazilian smoked sausages revealed PAH4 concentrations as high as 33.84 μg/kg, exceeding European Union (EU) regulatory thresholds for BaP of 2.0 μg/kg in smoked meat, fish and their products; and 83% of 205 commercially available Brazilian meat products were contaminated with at least one of nine monitored PAHs, with concentrations reaching 108.24 μg/kg (da Silva et al. 2024; da Silva et al. 2023; EC 2011). The type of food matrix is crucial, as evidenced by butter exhibiting higher PAH24 concentrations (mean 141.25 μg/kg) than margarine, with one sample exceeding the EU limit for PAH4 (Lan and Wu 2023).

Similarly, pan frying canola oil at 180°C for 15 min drastically increased BaP concentrations by 316.55% and total 15 PAHs by 443.32% (Ma et al. 2021). Positive correlations between saturated fatty acids like palmitic acid and specific PAHs (*t*_*s*_ = 0.79 for benzo[e]pyrene, *p* < 0.05) mechanistically connect fatty acid profiles to PAH formation, explaining why palm oil emitted the highest mass of particle‐bound PAHs during heating (Chiang et al. 2022). Critically, the incomplete combustion process not only yields parent PAHs but also generates derivatives including oxygenated (OPAHs) and nitrated (NPAHs) polycyclic aromatic hydrocarbons, which can exhibit 10 to 1000‐fold higher carcinogenic potency than their parent compounds (Zhang, Hu, et al. 2023). Upon ingestion, these toxicants can undergo metabolic activation to form DNA‐binding intermediates that initiate mutagenesis and carcinogenesis, linking chronic exposure to cancers of the gastrointestinal tract, liver, and lung (Gao et al. 2018; Venkatraman et al. 2024). Consequently, the appealing sensory qualities of thermally processed foods are intrinsically shadowed by this robust toxicological profile of incomplete combustion and protein pyrolysis.

## 4 Appealing Sensory Properties Co‐Form With Heat‐Induced Toxicants

The Maillard reaction and associated thermal processes create a profound paradox in modern food science: they simultaneously generate the sensory attributes consumers crave and a suite of hazardous chemical contaminants. This co‐formation of pleasant sensory compounds with HITs establishes a direct conflict between palatability and safety, where appealing flavors, colors, and textures effectively overshadow a toxicological footprint, complicating risk perception and management. As summarized in Table 2, the duality of thermal processing links the formation of desirable sensory properties with co‐formed toxicants in foods, providing a framework for understanding this inherent trade‐off between sensory quality and chemical safety.

**TABLE 2:** The duality of thermal processing: Linking the formation of desirable sensory properties with co‐formed toxicants in foods.

Sensory property	Sensory compound	Co‐formed toxicant	Formation factor	Quantitative link	References
Toasted nutty aroma	Alkylpyrazines; methylpyrazines	CEL	Shared α‐dicarbonyl intermediates; 150°C	Pyrazines dominate aroma profile; CEL exceeds CML	Shi et al. (2025)
Roasted nutty aroma	Alkylpyrazines	HAAs; acrylamide	Maillard reaction; > 120°C	2,5‐Dimethylpyrazine is a key contributor; acrylamide peaks at 150°C	Khalid et al. (2022)
Roasted coffee aroma	Alkylpyrazines	Acrylamide; HMF; furans	Coffee roasting (light→dark roast)	Acrylamide ↓; HMF ↑	Cascos et al. (2024)
Toasted flavor	Thiophenes; thiazoles	HAAs	Maillard reaction	Thiophenes yield roast notes	Park and Choi (2025)
Burned smoky aroma	4‐vinylguaiacol; methoxyphenols	PAHs	Wood smoking; fat pyrolysis	Methoxyphenols drive smoke aroma	Marušić Radovčić et al. (2016)
Burned‐sugar caramel notes	Furans (HMF, furfural)	HMF	Caramelization; > 150°C	HMF indicates thermal load; Furans give bitter flavor	Chan et al. (2025)
Caramel aroma	Strecker aldehydes	Acrylamide	Maillard reaction; > 110°C	Maillard yields acrylamide	Liu et al. (2022)
Golden‐brown color	Maillard melanoidins	Acrylamide	Deep‐fry potatoes; 180°C	Acrylamide forms via Maillard reaction	Karslıoğlu et al. (2024)
Grilled meat aroma	Lipid‐derived ketones; aldehydes	PAHs	Charcoal grilling; 260°C; lipid oxidation	PAHs from fat pyrolysis	Beriain et al. (2024)
Smoky char aroma	Aldehydes; alkanes	PAHs (BaP)	Charcoal grilling meat; 450°C	HMW PAHs ~20–28 μg/kg	Beriain et al. (2024)
Roasted, caramel flavor; brown color	Alkylpyrazines; furanones; HMF	Furan; HMF	Sugar caramelization; low pH	Aldehyde and 2‐Furfurylthiol generation; strong crust color correlation	Chan et al. (2025)
Caramel‐like, sweet aroma	4‐Hydroxy‐2,5‐dimethyl‐3 (2H)‐furanone	HMF	Medium‐temperature roasting; low pH	HMF indicates thermal load	Obando and Figueroa (2024)
Crispy golden texture	Crispy texture; Maillard melanoidins	Acrylamide; acrolein	Deep‐fry potato chips; > 120°C	Acrylamide and acrolein surge during processing	Santiago‐Mora et al. (2024)

Abbreviations: BaP, benzo[a]pyrene; CEL, Nε‐carboxyethyllysine; CML, Nε‐carboxymethyllysine; HAAs, heterocyclic aromatic amines; HMF, hydroxymethylfurfural; HMW, higher molecular weight; PAHs, polycyclic aromatic hydrocarbons.

### 4.1 Flavorful Aromas From Hazardous Reaction Pathways

The formation of flavorful aromas from hazardous heterocyclic compounds exemplifies this duality, as the same Maillard reaction pathways produce both desirable odors and potent toxicants. Alkylpyrazines contribute essential nutty and roasted notes to chocolate, coffee, and roasted meats, with their generation during cocoa fermentation and roasting being well documented (Echeverria et al. 2021; Mortzfeld et al. 2020). Disturbingly, these identical processes simultaneously generate HAAs, including PhIP and MeIQx (Gumus et al. 2024; Shi et al. 2025). Similarly, though some furan derivatives sparingly impart sweet, caramel‐like notes to baked products and canned meats, yet furan itself raises toxicological concern due to its carcinogenic potential (IARC 2023; Mirza Alizadeh et al. 2025). Thiophenes provide savory, meaty characteristics but belong to a broader class of heterocyclic organic compounds requiring careful examination (Eisentraeger et al. 2008). The biochemical pathways producing these appealing aromas are intrinsically linked to HAA formation through reactions among creatine, amino acids, and sugars at elevated cooking temperatures (Bellamri et al. 2021). Upon consumption, HAAs undergo metabolic activation via N‐hydroxylation, producing reactive heteroaryl nitrenium ions that form DNA adducts, a mechanism underlying their genotoxicity and multisite carcinogenicity in rodent models affecting the liver, colon, and prostate (Turesky and Le Marchand 2011). Biomonitoring studies confirm that humans efficiently convert dietary HAAs into these reactive intermediates, establishing a molecular link between well‐done meat consumption and elevated cancer risk in epidemiological studies (Bellamri et al. 2021). In roasted chicken, high‐temperature stages crucial for developing complex aroma profiles directly promote HAA formation, with studies quantifying significantly elevated PhIP, Norharman, and Harman levels compared to earlier processing stages (Zhang et al. 2022). Norharman and Harman act as co‐mutagenic agents that amplify the genotoxicity of other HAAs (Zhang et al. 2022). The very chemical processes creating nutty, roasted, and caramel‐like notes thus insidiously generate toxicants, effectively distracting the attention of the consumers from potential risks with flavorful aromas (El Hosry et al. 2025; Liu et al. 2022).

### 4.2 Desirable Colors Signal Potential Toxicant Presence

**FIGURE 2:**
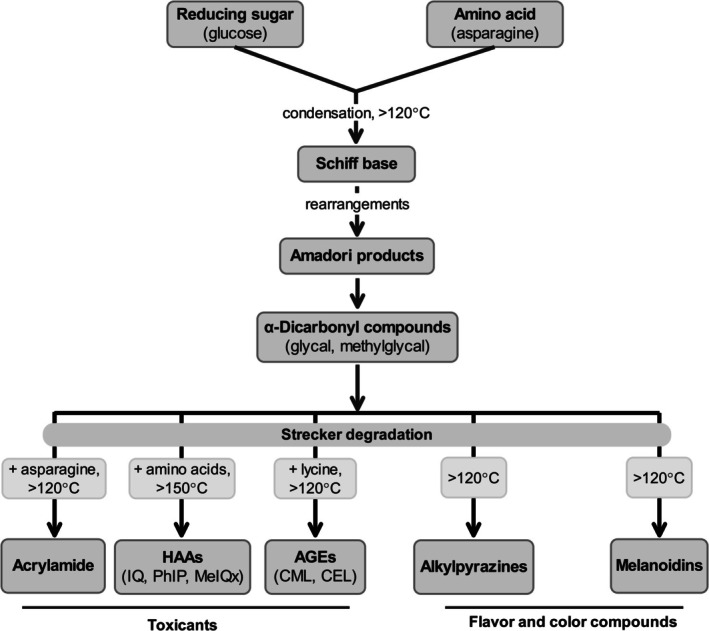
Schematic representation of the Maillard reaction as a branching pathway, highlighting its dual role in generating both desirable sensory compounds and toxicants. AGEs, advanced glycation end‐products; CEL, Nε‐carboxyethyllysine; CML, Nε‐carboxymethyllysine; HAAs, heterocyclic aromatic amines; IQ, 2‐amino‐3‐methylimidazo[4,5‐*f*]quinoline; MelQx, 2‐amino‐3,8‐dimethylimidazo[4,5‐*f*]quinoxaline; PhIP, 2‐amino‐1‐methyl‐6‐phenylimidazo[4,5‐*b*]pyridine.

The visually appealing golden‐brown hues that consumers actively seek in thermally processed foods directly signal the progression of the Maillard reaction, a complex cascade that simultaneously generates desirable sensory compounds and potentially harmful toxicants (Kaspchak et al. 2022). Figure 2 provides a schematic representation of this branching pathway, illustrating the dual role of the Maillard reaction in generating both desirable sensory compounds such as melanoidins and alkylpyrazines and toxicants like acrylamide, HAAs, AGEs, and PAHs. This non‐enzymatic browning culminates in melanoidins, high‐molecular‐weight nitrogenous polymers that serve as the principal chromophores responsible for the characteristic color of baked, roasted, and fried items (Wang et al. 2025). Research on fish skin collagen peptides demonstrates that more advanced reactions produce intense color development, with a marked color difference (ΔE) value reaching 31.78, which directly correlates with reaction progression and a significant reduction in free amino groups (Wu et al. 2025). Consumers intuitively rely on this progressive color development as a primary visual cue for assessing doneness. However, this same visual cue simultaneously signals the parallel formation of acrylamide, a neurotoxic and potentially carcinogenic compound generated from the reaction between asparagine and reducing sugars (El Hosry et al. 2025; Kaspchak et al. 2022). Ehling and Shibamoto established a strong, power law‐type correlation between acrylamide generation and browning color, revealing that under specific conditions (170°C for 30 min in a 1:3 asparagine: glucose system), acrylamide formation reaches 2629 μg/g of asparagine (Ehling and Shibamoto 2005). This correlation holds particular significance because acrylamide formation typically peaks after 20–30 min before undergoing elimination, whereas browning continues to intensify, meaning the desirable darkening often occurs after maximal toxicant formation. The consumer's instinctive reliance on this golden‐brown visual heuristic therefore creates a fundamental paradox: the sensory property that makes foods appealing simultaneously signals the presence of HITs formed through shared chemical pathways (Murata 2021; Qi et al. 2025).

### 4.3 Textural Changes Correlate With Toxicant Formation

The pursuit of desirable textural properties, particularly the crispiness and crunchiness that drive consumer acceptance of ultra‐processed foods (UPFs), inextricably links sensory appeal with the co‐formation of heat‐induced toxicants (Katsikari and Varela 2026). Achieving this characteristic mouthfeel in fried snacks necessitates high temperature regimes that simultaneously trigger deleterious chemical reactions. The process of submerging potato slices in oil at temperatures exceeding 120°C initiates rapid dehydration and structural breakdown of the cellular matrix, which yields the brittle architecture responsible for acoustic and tactile crispiness (Wang, McClements, et al. 2023). However, these same conditions constitute the *sine qua non* for acrylamide generation via the Maillard reaction between reducing sugars and asparagine, with kinetic models confirming that temperatures above 120°C exponentially drive the formation of the Schiff base intermediate (Mirza Alizadeh et al. 2025).

The requisite high heat for texturization also provokes lipid degradation pathways; when frying oils exceed 180°C, the dehydration of glycerol moieties generates acrolein, the pungent aldehyde that, while contributing to barbecued flavor nuances, poses significant cytotoxic hazards (Mirza Alizadeh et al. 2025). For proteinaceous fried commodities, the temperature window of 150°C–300°C that facilitates crust formation and crispiness concurrently drives the cyclization and condensation of amino acids and creatinine into HAAs, potent mutagenic compounds (Dong et al. 2020). Furthermore, incomplete combustion of organic matter during high‐temperature grilling or frying, particularly when fats pyrolyze directly over heat sources, produces PAHs, including the carcinogenic PAH4, which adsorb onto the dehydrated oil‐rich surface of the food (Yin et al. 2025). The morphology of a crispy crust, characterized by increased porosity and thickness developed during dehydration, is a key determinant for both desirable fracture properties and elevated oil uptake, which can act as precursor or carrier for toxicants (van Koerten et al. 2015). Therefore, a troubling synergy emerges in UPFs, where the same thermodynamic processes required to transform raw ingredients into sensorially satisfying crispy matrices inherently foster the multi‐contaminant burden that undermines the long‐term safety of thermally processed foods (Dangal et al. 2024; Menegassi and Vinciguerra 2025).

### 4.4 Consumer Preferences Drive Hazardous Production Practices

Consumer preferences drive hazardous production practices by compelling the food industry to optimize thermal processes that inherently generate HITs. This sensory‐driven demand exploits an intrinsic chemical entanglement: the Maillard and other related thermal reactions, responsible for desirable roasted aromas and golden‐brown colors, simultaneously produce processing contaminants like acrylamide and furan (Byrne 2020). Industry, responding to market expectations, inadvertently reinforces HIT formation. For instance, consumer liking drives the use of specific ingredients, such as glucose and whole egg in sponge cake formulations; under high‐temperature baking, these formulations preferentially generate furan through caramelization and lipid oxidation pathways (Cepeda‐Vázquez et al. 2019). Similarly, analysis of various potato chips demonstrates that consumer‐preferred products often contain higher relative levels of harmful aldehydes and ketones derived from frying (Zhang et al. 2025). This creates a self‐perpetuating feedback loop: the industry adapts its processes to meet sensory expectations for specific textures, colors, and flavors, which reinforces HIT formation, while consumers, guided by intuitive toxicology, continue selecting products based on positive sensory connotations rather than chemical risks detectable only below sensory thresholds (Siegrist and Hartmann 2020). These findings explain why European consumers, despite actively seeking food safety information, often misperceive chemical risks and maintain purchasing behaviors that reward hazardous production practices (Jaskiewicz et al. 2023). Importantly, emerging clean‐label trends, driven by consumer avoidance of artificial additives, catalyze a shift toward natural colorants and milder processing conditions. This movement disrupts the sensory toxicity cycle by promoting formulations that minimize acrylamide, HAAs, and PAHs while maintaining palatability (Mirza Alizadeh et al. 2025).

## 5 Health Implications of Heat‐Induced Toxicants

This section discusses evidence demonstrating how chronic dietary exposure to toxicants drives serious health conditions. We elucidate brief mechanisms by which these prevalent compounds initiate genotoxicity, neurotoxicity, and oxidative stress, thereby accelerating cancer risk and neurodegenerative pathologies across organ systems.

### 5.1 Carcinogenicity

The thermal processing of foods generates multiple carcinogenic toxicants, with the IARC providing critical hazard classifications that highlight their public health significance. Acrylamide, a Maillard reaction product formed primarily from asparagine and reducing sugars during high‐temperature cooking, is classified as a probable human carcinogen (Group 2A) (IARC 2023). Dietary exposure to acrylamide is widespread, with concentrations in French fries and potato chips ranging from 779 to 1299 and 211 to 3515 μg/kg, respectively, leading to an estimated intake of 0.4–1.9 μg/kg bw/day, with children experiencing the highest exposure levels (Başaran et al. 2023). A dose–response meta‐analysis from 2020 identified a positive association between dietary acrylamide intake and increased risk of endometrial and ovarian cancers, particularly among never‐smoking women (Hogervorst and Schouten 2022). The large prospective NutriNet‐Santé cohort further revealed a statistically significant increased breast cancer risk among premenopausal women with high acrylamide intake, reporting a hazard ratio of 1.40 (95% CI: 1.04–1.88), an association notably stronger for hormone receptor–positive tumors (Hogervorst and Schouten 2022). NHANES data analysis reinforced these findings, demonstrating that higher ultra‐processed food consumption correlates monotonically with a 9.1% increase in combined acrylamide and glycidamide hemoglobin adduct levels, establishing a plausible biological link between HIT exposure and health outcomes (Steele et al. 2023).

Simultaneously, HAAs, another critical class, demonstrate potent multisite carcinogenicity, predominantly forming in protein‐rich meats heated above 150°C (Dong et al. 2020). IARC evaluations identify specific HAAs as significant hazards; for instance, IQ (2‐amino‐3‐methylimidazo[4,5‐f]quinoline) is classified as probably carcinogenic to humans (Group 2A), while MeIQ, MeIQx, and PhIP are considered possibly carcinogenic (Group 2B) (IARC 2023). Epidemiological studies reveal positive associations between HAA exposure and cancer risk, with animal studies confirming these compounds target multiple organs including the breast, colon, liver, lung, and prostate (Bellamri et al. 2021). Notably, some HAAs exhibit 100‐ to 2000‐fold greater mutagenic potency than aflatoxin B1 and benzopyrene in certain assays (Aoudeh et al. 2024). Furthermore, among PAHs, benzo[a]pyrene is uniquely identified as a known human carcinogen Group 1, with its potent toxicity arising from metabolic activation that leads to DNA adduct formation (IARC 2023; Montano et al. 2025). These compounds often co‐occur in fried, roasted, and grilled meats, creating a potential cocktail effect that complicates risk assessment (Iammarino et al. 2024). Furan, a heterocyclic compound formed through thermal degradation of sugar, is considered possibly carcinogenic to humans (Group 2B) and is found in a broad range of heated foods such as canned and jarred foods like fruits in syrup and jams, jellies, soups, meats, caramel sauces, and sweetened condensed milk, with concentrations reported from 0 to over 6000 μg/kg, indicating a plausible genotoxic risk at dietary exposure levels (Mariotti et al. 2013; Mogol and Gökmen 2016). Current risk assessments for these HITs heavily rely on evidence from animal studies and exposure data, indicating that prolonged consumption even at low concentrations may pose a significant cancer risk (Batool et al. 2023; Batool et al. 2025). Consequently, the formation of these contaminants is intrinsically linked to desirable sensory properties, creating a complex challenge where appealing flavors and aromas in processed foods effectively disguise their underlying toxicological footprint.

### 5.2 Neurotoxicity

Beyond carcinogenicity, acrylamide stands out as a well‐characterized neurotoxin, with its deleterious effects documented in both occupational and dietary exposure settings, posing a risk of cumulative neuronal damage from regular intake of common foods like French fries and baked goods (Naji et al. 2025; Rajeh 2024). The neuropathological mechanism is multifaceted; it involves acrylamide forming irreversible adducts with presynaptic proteins, which directly inhibits neurotransmitter release and disrupts kinesin‐based axonal transport (Mollakhalili‐Meybodi et al. 2021; Rajeh 2024). This process promotes oxidative stress, mitochondrial dysfunction, and activates apoptotic pathways, culminating in distal axonopathy that manifests as peripheral neuropathy, ataxia, and cognitive impairment (Zhao et al. 2022). Critically, longitudinal human evidence corroborates this risk; a prospective cohort study found that each one‐standard‐deviation increase in dietary acrylamide was associated with a 7.698% decrease in Mini‐Mental State Examination score over 4 years and a significantly increased hazard ratio of 3.356 for poor cognition (Liu et al. 2017). The cumulative nature of this neurotoxicity is exacerbated by the slow turnover of affected neuronal proteins, leading to progressive dysfunction. Complementary experimental models reinforce these findings; rodent studies show chronic low‐dose exposure induces gait abnormalities and learning deficiencies, while a *Caenorhabditis elegans* model demonstrates significant locomotor impairment and degeneration of dopaminergic neurons (Ma et al. 2025; Zhao et al. 2022).

Moreover, emerging evidence now emphasizes neuroinflammation as a key mechanism driving this toxicity. Specifically, acrylamide significantly inhibits the expression and activity of key glycolytic enzymes such as triphosphate isomerase and 3‐phosphoglyceraldehyde dehydrogenase in BV2 microglial cells, leading to an accumulation of methylglyoxal (Wang et al. 2022). This carbonyl stress subsequently triggers the upregulation of the NF‐κB inflammatory signaling pathway, elevates the pro‐inflammatory factor TNF‐α, and induces further oxidative stress (Wang et al. 2022). Complementing these cellular findings, in vivo studies demonstrate that acrylamide intoxication (50 mg/kg for 11 days) robustly upregulates mRNA expression of neuroinflammatory cytokines TNF‐α, IL‐1β, and IL‐6 (*p* < 0.001) in rat brains, while simultaneously depleting antioxidants and increasing lipid peroxidation (Mirzavi et al. 2024).

Alarmingly, the public health concern is particularly acute for vulnerable subgroups; the margin of exposure for neurotoxicity dips below the safety threshold of 125 for high consumers (95th percentile) among children and adolescents, signaling a tangible concern given their higher dietary intake per body weight (Nematollahi, Kamankesh, Hosseini, Hadian, et al. 2020). Furthermore, the neurotoxic impact is potentially amplified by other food contaminants, as certain metals can inhibit acetylcholinesterase activity, thereby disrupting cholinergic signaling and compounding neurological deficits (Rajeh 2024).

### 5.3 Oxidative Stress and Inflammation

Heat‐induced toxicants, specifically acrolein and AGEs, instigate disease pathology primarily by hijacking the body's oxidative and inflammatory defense systems, thereby accelerating cardiovascular and neurodegenerative diseases. Acrolein, a highly reactive α,β‐unsaturated aldehyde classified as a Group 2A carcinogen, directly attacks cellular antioxidant capacity by adducting with and depleting glutathione and elevating reactive oxygen species (ROS), which subsequently activate pro‐inflammatory signaling via NF‐κB and the NLRP3 inflammasome; this cascade culminates in caspase‐1‐mediated maturation of IL‐1β and IL‐18, inducing pyroptosis (Xu et al. 2025; Zhang, Wang, et al. 2023). In the cardiovascular system, this cycle promotes endothelial dysfunction by impairing nitric oxide bioavailability and triggering inflammatory cell recruitment, a precursor to atherosclerosis and cardiomyopathy (Henning et al. 2017; Ling and Kuo 2018). Additionally, acrolein also induces systemic dyslipidemia by modifying circulating lipoproteins, further increasing atherosclerosis risk (Zirak et al. 2019). Simultaneously, AGEs from high‐temperature cooked foods engage their receptor (RAGE), activating NF‐κB and instigating pro‐inflammatory responses and ROS generation (Zirak et al. 2019). This chronic inflammation, or inflammaging, drives arterial stiffness and endothelial dysfunction (de Almeida et al. 2020). Clinically, circulating AGE levels correlate strongly with diabetic complication severity, such as retinopathy progression, underscoring this pathway's clinical relevance (Alfarhan et al. 2020). In the central nervous system, acrolein‐induced mitochondrial dysfunction amplifies ROS and releases damage‐associated molecular patterns, perpetuating neuroinflammation (Xu et al. 2025). Moreover, AGE‐RAGE signaling activates microglia, creating a cycle of mitochondrial oxidative stress and neuronal death implicated in Alzheimer's and Parkinson's diseases (Abadin et al. 2024; Rekatsina et al. 2020). These self‐perpetuating cycles of oxidative injuries and inflammations, therefore, represent a hidden health threat obscured by the sensory appeal of thermally processed foods.

### 5.4 Genotoxicity and Mutagenicity

The genotoxic and mutagenic impacts of heat‐induced food toxicants involve distinct mechanisms, with advanced methodologies like error‐corrected next‐generation sequencing (ecNGS) now providing high‐sensitivity detection of point mutations in human‐relevant models (Barutcu et al. 2025). For example, the Ames test reveals the mutagenic potential of furan through its reactive metabolite, cis‐2‐butene‐1,4‐dial (BDA); however, furan presents primarily clastogenic activity, inducing chromosomal damage and inter‐strand cross‐links rather than strong point mutagenicity (Russo et al. 2021). Conversely, HAAs are potent bacterial mutagens, but they universally require metabolic activation to exert genotoxicity. This activation proceeds via cytochrome P450‐mediated N‐hydroxylation followed by esterification through enzymes like N‐acetyltransferase 2 (NAT2) or sulfotransferase 1A1 (SULT1A1), ultimately forming reactive heteroaryl nitrenium ions that directly form DNA adducts (Barutcu et al. 2025; Bellamri et al. 2021). Similarly, acrylamide is metabolized by cytochrome P450 2E1 to its genotoxic epoxide, glycidamide, which readily forms pre‐mutagenic DNA adducts such as N7‐(2‐carbamoyl‐2‐hydroxyethyl)guanine (N7‐GA‐Gua) (Hölzl‐Armstrong et al. 2020). These adducts drive a distinct mutational signature characterized predominantly by A>T/T>A and A>G/T>C base substitutions, as confirmed in human TP53 knock‐in (Hupki) models where glycidamide exposure induced a TP53 mutant frequency of 9%, with mutations occurring at codons also found mutated in human tumors associated with acrylamide exposure (Hölzl‐Armstrong et al. 2020). Therefore, integrated testing strategies that combine ecNGS with cytogenetic endpoints thus provide a complete human‐relevant genotoxicity evaluation, strengthening the weight‐of‐evidence for these toxicants (Barutcu et al. 2025).

### 5.5 Cumulative Exposure Drives Chronic Disease Risk

Ultimately, ubiquitous in the modern diet, HITs such as acrylamide, PAHs, and acrolein pose a complex public health challenge, as long‐term, low‐dose cumulative exposure is increasingly linked to elevated chronic disease risk (Moradi et al. 2025). These compounds readily form in common foods like French fries, crisps, toasted bread, and coffee via high‐temperature processes such as frying, grilling, and roasting (Mirza Alizadeh et al. 2025). Crucially, co‐exposure to multiple classes of these toxicants is the norm rather than the exception in typical diets, and their combined effects on human health may be additive or synergistic, though difficult to characterize (Gloria et al. 2021). This concern is particularly acute for children, who experience higher dietary exposure per body weight and whose developing systems may be more vulnerable to toxic insults. Alarmingly, baby foods and common staples like cereals and biscuits can represent significant exposure sources, potentially elevating lifetime cancer risk early in life (Başaran et al. 2023). The mechanisms behind this risk are multifaceted; for instance, acrylamide is metabolized to the genotoxic derivative glycidamide, while PAHs form DNA adducts that can initiate mutations and cancer (Barul and Parent 2021).

Furthermore, individual susceptibility is modulated by genetic differences in toxicant metabolism and the modifying role of the gut microbiota (Yunusova et al. 2025). The pathological implications extend beyond cancer, as illustrated by research linking prenatal exposure to a mixture of PAHs to impaired fetal renal function, signified by a statistically significant decrease in estimated glomerular filtration rate (eGFR) of −1.09 mL/min/1.73 m^**2**^ per quartile increase in PAH mixture exposure (Hsu et al. 2024). Similarly, acrolein, identified as a uremic toxin, contributes to urothelial carcinomas in patients with chronic kidney disease (CKD), illustrating a vicious cycle where a HIT can exacerbate pre‐existing chronic conditions (Hong et al. 2020). Consequently, modern risk assessment paradigms must evolve beyond single‐component evaluation to fully consider the cumulative impacts of these HITs on chronic disease trajectories.

## 6 Detection and Quantification Methods for Heat‐Induced Toxicants

The analytical landscape for heat HITs is clearly dominated by advanced chromatographic and spectroscopic techniques, which provide the requisite sensitivity and specificity for precise quantification within complex food matrices. A summary of the primary analytical techniques, their key performance metrics, and applicable food matrices is provided in Table 3. For instance, chromatography coupled with mass spectrometry serves as the cornerstone, with methods consistently achieving detection limits ranging from micrograms per kilogram to milligrams per kilogram. A prime example is a robust 2025 μSPE–UHPLC–MS/MS method for 10 HAAs in plant‐based milk beverages, which achieved limits of detection (LOD) and quantification (LOQ) from 0.01 to 0.04 and 0.01 to 0.05 μg/L, respectively, backed by recovery rates of 84% to 100% (Mandelli et al. 2025). This method successfully quantified HAA concentrations from 0.09 to 13.66 μg/L, revealing significantly higher levels in thermally treated beverages with added sugar and protein (Mandelli et al. 2025). Similarly, an isotope dilution LC–MS/MS method for 12 HAAs and acrylamide demonstrated excellent precision with a relative standard deviation below 20%, accuracy between 71.8% and 119.1%, and detection limits under 3.1 ng/g for all analytes (Lee et al. 2015).

**TABLE 3:** Analytical methods for detection and quantification of selected heat‐induced toxicants in foods, with their quality parameters.

Toxicant	Matrix	Method	LOD (μg/kg)	LOQ (μg/kg)	Recovery/RSD (%)	References
Acrylamide	Potato chips	LC–MS/MS	10.00	36.00	81.00–100.00	Becker et al. (2025)
	Plantain chips	HPLC	4.00	10.00	99.00–105.00	Udomkun et al. (2021)
	Potato chips	RP‐HPLC‐DAD	0.27	3.25	—	Assefa et al. (2025)
	Snack foods	DLLME‐GC–MS	0.59	1.90	95.00	Kamankesh et al. (2021)
	Air‐fried fish/meat	GC–MS	0.63	2.08	—	Yoon et al. (2024)
	Cakes; biscuits	DLLME‐GC–MS	0.60	2.00	95.00	Nematollahi, Kamankesh, Hosseini, Ghasemi, et al. (2020)
Acrolein	Edible oils	DLLME‐GC–MS	0.05–0.10	0.15–0.30	95.00–110.00	Custodio‐Mendoza et al. (2020)
	French fries, potatoes, sausages	LC–MS/MS	0.14–1.73	0.43–5.24	82.12–119.30	Lim and Shin (2025)
	Sourdough, bread	HS‐SPME/GC–MS	1.21	4.05	5.86–6.43	Drakula et al. (2019)
Furan	Coffee products	GC–MS	1.50–6.00	5.00–20.00	2.20–10.40	Cao et al. (2022)
	Chocolate‐based products	HS‐SPME‐GC–MS	0.14–0.78	0.48–2.50	0.30–8.00	Alsafra et al. (2020)
	Dried corn; roasted peanuts	HS‐SPME‐GC/FID	0.54–3.50	1.80–12.00	67.00–106.00	Masite et al. (2022)
	Canned meat, puree, paste	GC–MS/MS	0.001–0.048	0.003–0.675	2.00–20.00	Tsao et al. (2022)
	Canned meat, jam, fruits	HS‐SPME‐GC–MS/MS	0.002–1.07	0.006–3.570	3.00–15.00	(Huang et al. 2022)
HAAs	Crispy fried pork	UPLC‐MS/MS	0.030–0.122	0.122–0.488	74.50–116.10	Z. Hu et al. (2025)
	Plant‐based milk	μSPE‐UHPLC–MS/MS	0.01–0.04	0.01–0.05	84.00–100.0	Mandelli et al. (2025)
	Chicken meat	HPLC	0.002–0.005	0.005–0.016	71.69–94.74	Gumus and Kizil (2022b)
	Deep‐fried meatballs	HPLC	2.50–20.00	2.50–20.00	> 70.00	Guzel et al. (2024)
	Meatballs	HPLC‐DAD	0.004–0.025	0.013–0.085	28.94–82.15	Uzun and Oz (2021)
PAHs	Baby food	GC–MS/MS	0.019–0.036	0.06–0.11	73.10–110.70	Ingegno et al. (2024)
	Barbecued meat	GC–HRMS	0.03–0.17	—	72.00–109.00	Zastrow et al. (2021)
	Air‐fried fish/meat	GC–MS	0.07–0.09	0.21–0.28	—	Yoon et al. (2024)
	Grilled chicken	QuEChERS‐GC–MS	0.03–0.20	0.10–0.60	83.47–110.83	Lim and Shin (2025)
	Fish, canned fish	GC–MS	0.07–0.18	0.21–0.54	80.00–120.00	Kim et al. (2022)

Abbreviations: DLLME‐GC–MS, automated dynamic liquid–liquid microextraction gas chromatography with mass spectrometry; GC–HRMS, gas chromatography with high resolution magnetic sector; GC–MS, gas chromatography with mass spectrometry; GC–MS/MS, gas chromatography tandem mass spectrometry; HPLC, high‐performance liquid chromatography; HPLC‐DAD, high‐performance liquid chromatography with diode‐array detection; HS‐SPME/GC–MS, headspace solid‐phase microextraction gas chromatography with mass spectrometry; HS‐SPME‐GC/FID, headspace solid‐phase microextraction gas chromatography with flame ionization detector; HS‐SPME‐GC–MS/MS, headspace solid‐phase microextraction gas chromatography tandem mass spectrometry; LC–MS/MS, liquid chromatography tandem mass spectrometry; LOD, limit of detection; LOQ, limit of quantification; QuEChERS‐GC–MS, quick, easy, cheap, effective, rugged and safe gas chromatography with mass spectrometry; RP‐HPLC‐DAD, reversed‐phase high‐performance liquid chromatography with diode‐array detection; RSD, relative standard deviation; UPLC‐MS/MS, ultra‐performance liquid chromatography tandem mass spectrometry; μSPE‐UHPLC–MS/MS, miniaturized solid phase extraction ultra‐performance liquid chromatography tandem mass spectrometry.

In contrast to HAAs, analytical protocols for PAHs, analytical protocols must meet stringent regulatory criteria, including recovery rates of 50%–120% and LODs of ≤ 0.30 ng/g (Chen, Inbaraj, and Hsu 2022). While GC–MS and HPLC with fluorescence detection are conventional workhorses, a novel 2024 approach using proton transfer reaction mass spectrometry (PTR–MS) coupled with a CHARON particle inlet achieved ultrasensitive, direct quantification of nine condensed PAHs in aerosols, with sub‐nanogram per cubic meter LOD ranging from 19 to 46 pg/m^**3**^ (Reinecke et al. 2024). These sophisticated methods expertly handle challenging, lipid‐rich matrices through rigorous sample preparation, including solid phase extraction cleanup, to mitigate interfering compounds. Consequently, the continued refinement of these chromatographic and spectroscopic techniques ensures the generation of precise data that is fundamental for regulatory compliance and proactive public health protection against the toxicological threat of processed foods.

## 7 Strategic Mitigation and Control of the Heat‐Induced Toxicants

### 7.1 Acrylamide

Strategic mitigation of the probable human carcinogen acrylamide necessitates a multi‐pronged approach targeting its precursors, asparagine and reducing sugars, across the entire food production chain. At the raw material level, selecting low asparagine crop varieties is fundamental; for example, agronomic research has successfully used CRISPR/Cas9 gene editing to knockout the TaASN2 gene in wheat, directly reducing this primary precursor in grains (Oddy et al. 2020). Additionally, compositional interventions effectively reconfigure the food matrix to impede the Maillard reaction. Incorporating lactic acid bacteria and sourdough consortia during fermentation metabolizes asparagine and lowers pH, achieving dramatic reductions of up to 79.6% in rye crispbread (ÜNal 2025). Likewise, adding glycine, specific cations like Mg^**2+**^ and Ca^**2+**^, or acidifying agents such as citric acid suppresses acrylamide formation by altering reaction dynamics (Negoiță et al. 2022; Sarion et al. 2021; Timilsena et al. 2013). Antioxidants including vitamins C and E may further interrupt radical formation pathways, adding another layer of control (Mohdaly et al. 2022).

Strategic pre‐processing forms a critical second pillar, particularly for potato products. Soaking raw potato strips in water for 15–30 min leaches precursor sugars, resulting in a statistically significant acrylamide reduction (*p* = 0.029) during frying (Navruz‐Varlı and Mortaş 2024). In contrast, blanching offers a more robust industrial scale hydrothermal treatment for precursor removal, while the targeted biochemical action of the enzyme asparaginase hydrolyzes asparagine into aspartic acid, thereby eliminating the key substrate (Israilides and Theodoros 2015; Kumari et al. 2022). Moreover, fermentation with specific microbes consumes these sugars, and physical pre‐treatments like pre‐drying modify cooking dynamics by reducing surface moisture and subsequent frying time (Israilides and Theodoros 2015; Mirza Alizadeh et al. 2025). These diverse mitigation strategies at the ingredient and process level are highlighted in Table 4.

**TABLE 4:** Diverse mitigation strategies for major heat‐induced toxicants.

Toxicant	Mitigation strategy	Food type	Mode of action	Efficiency	Limitation	References
Acrylamide	Air frying (170°C, 8 min)	French fries	Minimizes oil absorption	50% reduction vs. deep frying	Inferior sensory profile	Ahmed et al. (2023)
	Industrial roasting (203°C;–205°C; 14 min)	Roasted coffee	Thermal degradation exceeds formation rate	Achieves ~300 μg/kg	Requires precise temperature control	Bertuzzi et al. (2020)
	Asparaginase treatment (0.15÷12 U/100 g of flour)	Pizza base	Precursor (asparagine) depletion via enzymatic hydrolysis	Up to 89% reduction	Alters dough rheology at high doses	Covino et al. (2023)
	Lactic acid fermentation ( *Lactobacillus plantarum* ; 37°C; 120 min)	French fries	Reduces free reducing sugar content	53.96% reduction	Imparts sour taste	Verma et al. (2022)
	Spice addition (Turmeric, 0.5%)	Beef steak	Antioxidants inhibit Maillard reaction pathways	56.81% reduction	Flavor compatibility essential	Kwon et al. (2025)
	Tea polyphenols (gallocatechin, 0.5 g/mol Asn)	Maillard model system	Scavenges persistent Maillard free radicals	Up to 55.7% reduction	Non‐dose dependent efficacy; structural specificity	Ye et al. (2024)
	Citric acid soaking (0.05%–1.00% conc.; 30 min)	French fries	Protonates asparagine amine group; blocks Maillard reaction	Near 100% inhibition	Imparts sour taste	Negoiță et al. (2022)
	Hot‐water blanching (70°C;–80°C, 5–15 min)	French fries	Leaches precursors via diffusion	Up to 97% reduction	Causes texture softening	Negoiță et al. (2022)
	Microwave baking (700 W)	Biscuits	Limits Maillard reaction temperature	Up to 68% reduction	Harder texture, surface irregularities	Al‐Ansi et al. (2019)
	Ultrasound pretreatment (35 kHz, 95.2 W/kg, 42°C)	Fried potatoes	Cavitation extracts precursors; increases moisture	Up to 83% reduction	Potential cell damage at high intensity	Antunes‐Rohling et al. (2018)
	Vacuum frying: 120°C; and 140°C, 10 and 15 min	French fries	Lowers reaction temperature	Up to 81% reduction	Specialized equipment needed	Verma et al. (2023)
Acrolein	Avoid overheating oil	Fried foods, frying oils	Prevents thermal hydrolysis of triglycerides	Formation increases with temperature > 160°C	Lower cooking temperatures may affect texture	Jiang et al. (2022)
	Avoid overusing oil	Fried foods, frying oils	Limits glycerol and PUFA degradation; thus, reduces accumulated degradation products	Prevents 10‐fold increase (reheated oil)	Requires frequent oil replacement	Jiang et al. (2022)
	Select healthier oils	General frying	Lower polyunsaturated fatty acids reduces precursor content	Varies by oil type	Flavor compatibility	Vignesh et al. (2024)
	Replace leavening agent	Biscuits	Avoids ammonia generation	Up to 87.2% reduction in related toxicant	Can alter final product size	Lo Faro et al. (2022)
HAAs	Blueberry extract marinade (0.5%)	Beef steak	Quenches free radicals; blocks HAA precursors	Up to 100% inhibition	Pro‐oxidant effects at certain concentrations	Gumus and Kizil (2022a)
	Bilberry extract marinade (0.25%–1%)	Chicken thigh	Antioxidant compounds mitigate radical formation	50.5%–99.3% reduction	Efficacy decreases at higher temperatures	Gumus and Kizil (2022b)
	Reishi extract addition (0.25% w/w)	Deep‐fried meatballs	Antioxidants scavenge free radical intermediates	Up to 100% inhibition	Flavor compatibility, concentration‐dependent effects	Guzel et al. (2024)
	Propolis marinade (0.25%–0.5% w/w)	Pan‐fried chicken	High phenolic content quenches reactive radicals	Up to 91.4% reduction	Potential allergen, strong characteristic flavor	Gumus and Kizil (2023)
	Antioxidant: Resveratrol (0.25%)	Ground beef patties	Scavenges phenylacetaldehyde intermediate; Electrophilic substitution	71% PhIP inhibition; Total MeIQ inhibition	Inconsistent organoleptic acceptance	Meurillon et al. (2020)
PAHs	Garlic essential oil (0.002–0.006%)	Charcoal‐grilled sausage	Scavenges free radicals via sulfides	Up to 57.1% reduction	Potential flavor alteration	Hu et al. (2021)
	Dried fruits (200 g/kg)	Roasted pork loin	Antioxidants scavenge free radicals	100% BaP inhibition	Flavor compatibility concerns	Bulanda and Janoszka (2023)
	Honey‐spices marination	Grilled beef satay	Forms protective surface barrier layer	Up to 28% PAH reduction	May increase total PAHs significantly	Nor Hasyimah et al. (2022)
	Shallot juice marination (10%–20% concentration)	Grilled goat satay	Antioxidants deposit into meat	BaP and BaA reduced to undetectable	Shallot availability concern	Saputro et al. (2022)
	*Jalapeno* pepper extract marinades	Grilled pork neck	Scavenges free radicals effectively	Up to 95% reduction	Strong spice flavor	Onopiuk et al. (2022)
	Green tea extract (25 g/kg)	Roasted duck skin	Scavenges free radical intermediates	75.8% BaP inhibition	Potential flavor alteration	Shen et al. (2022)
	Lower smoking temperature	Pork sausages	Reduces precursor pyrolysis intensity	3‐fold lower PAH4 levels	May alter product sensory profile	Racovita et al. (2020)
	Air frying (vs. deep‐fat frying)	Fried chicken meat	Limits oil‐mediated lipid pyrolysis	~31% lower PAHs	Alters final product texture	Lee et al. (2020)
	Gamma irradiation (5 kGy)	Smoked guinea fowl meat	Degrades high molecular weight PAHs	Up to 100% BaP reduction	Requires specialized facilities	Otoo et al. (2022)

Abbreviations: BaA, benz[a]anthracene; BaP, benzo[a]pyrene; HAAs, heterocyclic aromatic amines; MeIQ, 4‐methyl‐2‐amino‐3‐methylimidazo‐[4,5‐f]quinoline; PAHs, polycyclic aromatic hydrocarbons; PhIP, 2‐amino‐1‐methyl‐6‐phenylimidazo[4,5‐b]pyridine; PUFA, polyunsaturated fatty acids.

**FIGURE 3:**
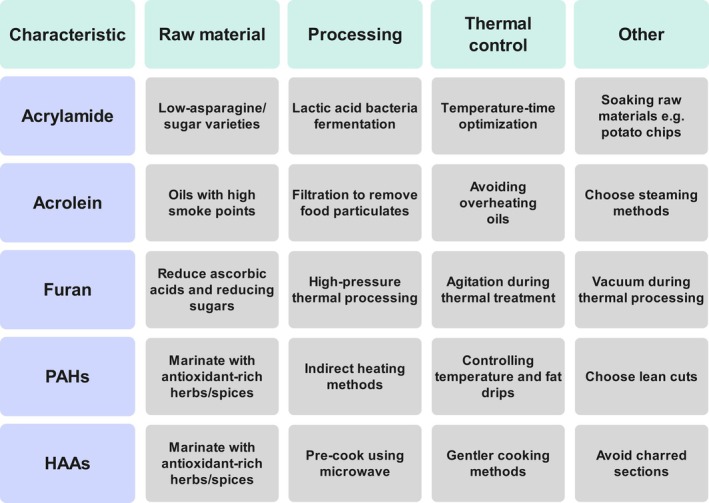
A multi‐faceted intervention framework targeting major heat‐induced toxicants across food processing stages. HAAs, heterocyclic aromatic amines; PAHs, polycyclic aromatic hydrocarbons.

The final mitigation tier involves rigorous optimization of thermal processing parameters, since acrylamide formation initiates above 120°C. Applying milder temperature time combinations directly disrupts this pathway; reducing frying temperature from 190°C to 175°C substantially decelerates formation kinetics, a phenomenon explained by its high activation energy (*E*_***a***_) of 10–160 kJ/mol (Perera et al. 2021). Consequently, selecting cooking methods like boiling, steaming, or microwaving that employ lower thermal loads provides significant mitigation for many foods (Vidhya et al. 2024). Where frying is indispensable, innovative low‐temperature vacuum frying minimizes acrylamide while preserving texture (Verma et al. 2023). Notably, a critical and universally applicable control point is visual guidance; achieving a golden yellow color in potato and cereal products instead of a dark brown hue is essential, as the latter strongly correlates with heightened acrylamide content (Boyaci Gunduz 2023). In summary, this integrated strategy, spanning cultivar selection, pre‐processing biochemistry, and thermal control, is illustrated in Figure 3 and provides a comprehensive framework for mitigating the toxicological profile of acrylamide and other HITs in processed foods.

### 7.2 Acrolein

Management of acrolein, a pervasive and highly toxic α,β‐unsaturated aldehyde, necessitates precise interventions during thermal food processing targeting its formation pathways in heated lipids (Jiang et al. 2022). Adopting precise thermal control by avoiding overheating oils beyond their smoke point is foundational; acrolein generation escalates dramatically during the thermal degradation of triglycerides, with heated rapeseed oil containing levels up to 198 mg/kg (Jiang et al. 2022). Furthermore, selecting oils with high smoke points and stable fatty acid profiles directly influences acrolein yield. Recent research delineates that acrolein formation arises not only from traditional radical oxidation but also via singlet oxygen (^**1**^O_**2**_) photo oxidation, with specific hydroperoxide isomers from ALA producing double the acrolein compared to other isomers, thus mandating protection of oils from light during storage (Kato et al. 2022). Operationally, implementing filtration to remove food particulates from used oil is a crucial operational step, as burned debris catalyzes further oxidative decomposition and acrolein formation (Mirza Alizadeh et al. 2025). Finally, prompt disposal of darkened or repeatedly used cooking oil is imperative, as prolonged use leads to accumulated degradation products and a marked increase in acrolein concentration. Studies reveal reheating oil can cause a 10‐fold increase in acrolein content, with frying oils showing levels between 7.4 and 198 mg/kg, thereby continuously elevating the toxicological load in prepared foods (Chang et al. 2022). Collectively, these targeted physical mitigation strategies effectively complement emerging biochemical approaches, offering a multi‐faceted defense to reduce dietary acrolein intake and its associated health risks (Abedi et al. 2023).

### 7.3 Furans and Furan Derivatives

Implementing mitigation and control of furans and their derivatives necessitates a multi‐faceted approach that strategically targets formation pathways through thermal parameter alterations and food formulation modifications (Oh et al. 2024). A primary intervention involves optimizing thermal processing conditions, as furan generation is intensely time‐ and temperature‐dependent during Maillard reactions, carbohydrate caramelization, and thermal oxidation of ascorbic acid and polyunsaturated fatty acids (Mirza Alizadeh et al. 2025). Innovative technologies like high‐pressure thermal processing at 600 MPa and 115°C significantly reduce furan and methylfuran levels in infant meals by minimizing thermal load, thereby ensuring microbiological safety while curtailing toxicant formation (Sandjong Sayon et al. 2025). Concurrently, leveraging the high volatility of these compounds presents a potent tactic; for example, stirring vegetable‐based infant meals for 240 s after reheating drives a reduction of up to 66.3% in combined furan concentrations by facilitating volatilization (Sandjong Sayon et al. 2024). Furthermore, modifying food composition by reducing key precursors such as ascorbic acid and PUFAs or adding competitive reactants diverts reaction pathways away from furan formation (Oh et al. 2024). However, such reduction must be carefully balanced, as ascorbic acid is an essential micronutrient vital for immune function and iron absorption, while PUFAs are critical for neonatal neural development and the regulation of inflammatory responses. The efficacy of these interventions is validated by advanced analytical methods; solid‐phase microextraction Arrow coupled with GC–MS/MS now enables simultaneous determination of furan and up to 10 derivatives with limits of quantitation as low as 0.006–3.571 ng/g, which is crucial for accurate exposure assessment (Oh et al. 2024). Risk characterization underscores the urgency of these strategies, as margin of exposure values for infants and toddlers often fall between 331 and 6354, well below the safety threshold of 10,000, indicating a persistent health concern from residual concentrations even after mitigation (Minorczyk et al. 2023). Therefore, a comprehensive control framework integrating novel industrial processing with clear consumer guidance on preparation practices is essential to effectively minimize the toxicological footprint of these HITs.

### 7.4 Polycyclic Aromatic Hydrocarbons

Reduction of PAHs necessitates a multi‐faceted approach targeting processing parameters, fuel selection, and biochemical interventions. Crucially, re‐engineering heat application by implementing indirect heating methods physically separates food from smoke, significantly reducing the direct deposition of airborne PAHs (Ciecierska et al. 2025; Iko Afé et al. 2020). Specifically, processors must avoid direct food contact with open flames during grilling, as this induces pyrolysis of organic matter exceeding 200°C, a primary PAH generation route (Das et al. 2023). Equally important is controlling temperature and preventing fat from dripping onto heat sources; this ignition of dripped fat generates PAH‐laden smoke that coats food, a major contamination mechanism (Das et al. 2023).

Beyond thermal management, fuel selection presents another control point; hardwoods over softwoods and charcoal carbonized at high temperatures, approximately 750°C, undergo more complete combustion, consequently generating less PAH, with emissions dropping from 7.26 to 0.78 μg/g (Chaemsai et al. 2016). Furthermore, pre‐heating charcoal until it glows, for instance, for 5 h at 650°C, drastically cuts PAH release from 19.86 to 0.69 μg/g (Chaemsai et al. 2016).

In parallel, biochemical interventions, such as marination with antioxidant‐rich herbs and spices, serve to quench free radicals and inhibit PAH formation; *jalapeno* pepper marinade achieved a 95% reduction in Σ12PAHs, while specific “universal” marinades reduce heavy PAHs by 24%–29% (Ciecierska et al. 2025; Cordeiro et al. 2020). Technological adoption, including electric grills over direct charcoal or using aluminum trays to catch fat drips, substantially lowers contamination, with low‐density polyethylene packaging adsorbing and reducing BaP by 73% (Ciecierska et al. 2025; Kim et al. 2021). Biological detoxification using lactic acid bacteria, such as *Lactobacillus brevis* TDA, demonstrates considerable PAH‐binding efficiency, up to 67.83% for BaP, via adsorption to cell walls (Yousefi et al. 2021; Zhao et al. 2013). Finally, a simple yet effective post‐processing step involves the removal of charred portions from grilled meat, as PAHs predominantly concentrate on the exterior surface, thereby directly reducing the ingested toxicant load (Barbosa et al. 2022; Das et al. 2023).

### 7.5 Heterocyclic Aromatic Amines

The implementation of strategic mitigation for HITs, specifically HAAs, leverages natural antioxidant interventions which disrupt key formation pathways through free radical scavenging and carbonyl trapping. Research demonstrates that marination with plant extracts like *Vaccinium myrtillus* L. extract significantly suppresses HAA levels in chicken thigh meat, achieving total HAA inhibition rates of 86.7% to 99.3% at 150°C and 50.5% to 98.1% at 200°C, with efficacy being both concentration‐ and temperature‐dependent (Gumus and Kizil 2022b). Similarly, blueberry extract outperformed propolis extract in pan‐fried beef, achieving up to 100% total HAA inhibition, a superiority attributed to its higher total phenolic content (345.6 mg GAE/g) and antioxidant status (346.0 mmol TE/kg) (Gumus and Kizil 2022a).

Critically, the method of application critically influences outcomes; in deep‐fried meatballs, incorporating reishi mushroom extract (RME) via an addition method proved significantly more effective than surface spreading, with 0.25% RME being most efficacious despite 1% RME providing superior lipid oxidation control, indicating HAA formation involves mechanisms beyond lipid peroxidation (Guzel et al. 2024). A common limitation, however, is the potential for pro‐oxidant effects, where higher concentrations of RME or artichoke extract can inadvertently promote specific HAA formation like PhIP (Guzel et al. 2024). This complexity extends to other natural additives; for instance, while 1% bitter melon extract reduced total HAAs by up to 69.9% (Gumus et al. 2024), and propolis extract decreased them by 41.2%–91.4% (Gumus and Kizil 2023), onion water extracts increased total HAA content, primarily elevating MeIQ (Nuray and Oz 2019). The efficiency and limitations of these various natural interventions for HAAs are consolidated in Table 4.

Consequently, a more targeted carbonyl trapping approach using highly nucleophilic phenolics like phloroglucinol proves exceptionally effective, reducing PhIP, MeIQx, IQ, and MeIQ formation in beef patties by 96%, 88%, 76%, and 83%, respectively, a principle that translates practically to over 90% HAA inhibition with apple or pear juice immersion (Hidalgo and Zamora 2023). Alternatively, direct modulation of the Maillard reaction by adding free amino acids like tryptophan (0.50%) inhibits total HAA formation by 93% (Linghu et al. 2020). Complementarily, systematic spice screening identifies ginger and clove as potent inhibitors, reducing total HAAs by 86.41% and 84.75%, respectively, with a significant inverse correlation between total free phenolic content and HAA levels (Wang, Wang, et al. 2023). Molecular modeling further refines ingredient selection, identifying resveratrol and carvacrol as highly effective, with oregano (rich in carvacrol) totally inhibiting MeIQ (Meurillon et al. 2020).

Moreover, the cooking medium itself modulates HAA yields, as evidenced by total HAA content in deep‐fat‐fried meatballs varying significantly from 30.43 ng/g in hazelnut oil to 43.71 ng/g in commercially mixed oil (Ekiz and Oz 2019). Ultimately, the most fundamental control strategy involves selecting gentler cooking methods; deep fat frying generates the highest total HAA levels in turkey meat (52.34 ng/g), followed by oven cooking, whereas boiling and microwaving result in significantly lower amounts, often below 2.5 ng/g, as HAA formation is minimal at temperatures below 100°C (Oz et al. 2016; Oz and Yuzer 2017). These findings collectively highlight that successful HAA mitigation is a multifaceted endeavor, highly dependent upon the synergistic optimization of antioxidant source, application method, concentration, cooking temperature, and food matrix composition.

## 8 Future Research Outlook

Substantial knowledge gaps persist regarding HITs, demanding a coordinated research agenda. Future work should first focus on elucidating molecular mechanisms through the application of isotope labeling and computational modeling to delineate precise formation pathways and kinetics for major toxicants like acrylamide and HAAs (Barutcu et al. 2025; Hölzl‐Armstrong et al. 2020). Identifying these critical branching points will reveal strategic interventions. In parallel, research should prioritize the development of integrated processing solutions that combine non‐thermal technologies in hurdle approaches, subsequently engineering these systems for viable industrial scale‐up to enhance efficiency and economic viability (Mirza Alizadeh et al. 2025).

Concurrently, a key objective is to assess the long‐term health impacts of low‐dose, cumulative exposure to HIT mixtures. This requires large‐scale epidemiological studies that track chronic disease trajectories, coupled with the development of robust biomarkers for both exposure monitoring and effect assessment (Hsu et al. 2024; Moradi et al. 2025). Furthermore, it is crucial to optimize mitigation strategies for sensory acceptance. This involves systematically screening and validating natural inhibitors, such as specific antioxidants and hydrocolloids, to ensure they effectively reduce toxicants without compromising the consumer‐desired flavors and textures that drive product acceptability (Byrne 2020; Wang, Wang, et al. 2023).

Finally, the scientific community must standardize advanced analytical methodologies. Validating and harmonizing methods like UHPLC–MS/MS across laboratories will ensure robust monitoring, a process that should be supported by creating certified reference materials for key toxicants to guarantee data comparability and regulatory reliability (Chen, Inbaraj, and Hsu 2022; Mandelli et al. 2025). Collectively, addressing these priorities will forge a multidisciplinary path toward safer thermally processed foods without sacrificing their sensory appeal.

## 9 Conclusion

The central paradox of thermal food processing lies in the biochemical inseparability of sensory appeal from toxicological risk. The Maillard reaction, caramelization, and lipid degradation generate desirable flavors and golden‐brown hues but simultaneously produce hazardous compounds such as acrylamide, HAAs, PAHs, and AGEs. Chronic dietary exposure to these heat‐induced toxicants is associated with oxidative stress, inflammation, and genotoxicity, mechanistically linked to carcinogenicity, neurotoxicity, and metabolic disorders. This duality undermines public health because the sensory cues consumers actively seek, such as roasted aromas and crispy textures, directly signal the presence of these hazardous contaminants. However, strategic interventions across the food production chain offer viable solutions. Raw material selection, precursor depletion via enzymatic or fermentative processes, and optimized thermal parameters can substantially reduce toxicant loads. Furthermore, natural antioxidant‐rich marinades and emerging non‐thermal technologies effectively suppress toxicant formation while preserving desirable sensory properties. Analytical advances now enable precise quantification of these contaminants, supporting rigorous risk assessment and regulatory compliance. Ultimately, reconciling this intrinsic conflict requires an integrated approach that simultaneously mitigates toxicant formation and maintains consumer acceptance. Future research must therefore prioritize molecular elucidation of formation pathways, validation of synergistic mitigation strategies, and development of robust biomarkers for cumulative exposure assessment. Only through such multidisciplinary efforts can the food industry deliver products that are both sensorially satisfying and demonstrably safe for long‐term human health.

## Author Contributions

**Joachim Dotto Matondo:** conceptualization, investigation, visualization, writing – original draft. **Abdulsudi Issa‐Zacharia:** writing – review and editing, visualization.

## Funding

The authors have nothing to report.

## Ethics Statement

The authors have nothing to report.

## Conflicts of Interest

The authors declare no conflicts of interest.

## Data Availability

Data sharing is not applicable to this article as neither new data were created nor analyzed.
